# Severe asthma is associated with a remodeling of the pulmonary arteries in horses

**DOI:** 10.1371/journal.pone.0239561

**Published:** 2020-10-22

**Authors:** Serena Ceriotti, Michela Bullone, Mathilde Leclere, Francesco Ferrucci, Jean-Pierre Lavoie

**Affiliations:** 1 Department of Clinical Sciences, Faculty of Veterinary Medicine, University of Montreal, Saint-Hyacinthe, Quebec, Canada; 2 Department of Health, Animal Science and Food Safety, Università degli Studi di Milano, Milano, Italy; Forschungszentrum Borstel Leibniz-Zentrum fur Medizin und Biowissenschaften, GERMANY

## Abstract

Pulmonary hypertension and *cor pulmonale* are complications of severe equine asthma, as a consequence of pulmonary hypoxic vasoconstriction. However, as pulmonary hypertension is only partially reversible by oxygen administration, other etiological factors are likely involved. In human chronic obstructive pulmonary disease, pulmonary artery remodeling contributes to the development of pulmonary hypertension. In rodent models, pulmonary vascular remodeling is present as a consequence of allergic airway inflammation. The present study investigated the presence of remodeling of the pulmonary arteries in severe equine asthma, its distribution throughout the lungs, and its reversibility following long-term antigen avoidance strategies and inhaled corticosteroid administration. Using histomorphometry, the total wall area of pulmonary arteries from different regions of the lungs of asthmatic horses and controls was measured. The smooth muscle mass of pulmonary arteries was also estimated on lung sections stained for α-smooth muscle actin. Reversibility of vascular changes in asthmatic horses was assessed after 1 year of antigen avoidance alone or treatment with inhaled fluticasone. Pulmonary arteries showed increased wall area in apical and caudodorsal lung regions of asthmatic horses in both exacerbation and remission. The pulmonary arteries smooth muscle mass was similarly increased. Both treatments reversed the increase in wall area. However, a trend for normalization of the vascular smooth muscle mass was observed only after treatment with antigen avoidance, but not with fluticasone. In conclusion, severe equine asthma is associated with remodeling of the pulmonary arteries consisting in an increased smooth muscle mass. The resulting narrowing of the artery lumen could enhance hypoxic vasoconstriction, contributing to pulmonary hypertension. In our study population, the antigen avoidance strategy appeared more promising than inhaled corticosteroids in controlling vascular remodeling. However, further studies are needed to support the reversibility of vascular smooth muscle mass remodeling after asthma treatment.

## Introduction

Severe equine asthma (also known as Heaves and Recurrent Airway Obstruction) is a chronic, non-infectious inflammatory lower airway disease, characterized by recurrent episodes of airway obstruction induced by exposure to environmental antigens (including fungi, hay mites and endotoxins) [1–3]. Pulmonary hypertension and reversible *cor pulmonale* are reported as clinical complications of severe equine asthma [4–6]. Diffuse airway obstruction increases regions of alveolar dead space. The resulting ventilation/perfusion mismatch impairs normal pulmonary gas exchanges, leading to alveolar hypoxia and eventually hypoxemia [7, 8]. Chronic hypoxia and hypoxemia exacerbate sustained hypoxic pulmonary vasoconstriction, increasing pulmonary vascular resistance and contributing to pulmonary hypertension onset and progression [9]. In asthmatic horses, airway obstruction was recently shown to induce pulmonary hypertension with right ventricular structural and functional alterations [10]. Their pulmonary artery pressure, measured by invasive right heart catheterization, inversely correlates with arterial oxygen tension, suggesting a significant role of hypoxic pulmonary vasoconstriction in increasing pulmonary vascular resistance and subsequently the mean pulmonary artery pressure [10, 11]. However, pulmonary hypertension is only partially reversed by oxygen administration and asthmatic horses have a significantly higher pulmonary artery pressure compared to healthy horses, even during disease remission [10, 11]. Therefore, other factors likely contribute to pulmonary hypertension development onset in asthmatic horses.

In experimental rodent models, chronic hypoxia, hypoxemia and allergic airway inflammation induce pulmonary vascular remodeling that mainly involves small muscular pulmonary arteries [12–18]. Pulmonary vascular remodeling is also well documented in chronic inflammatory respiratory disorders in humans such as chronic obstructive pulmonary disease (COPD) where it is recognized as a determining factor in the onset of pulmonary hypertension and *cor pulmonale* [19, 20]. Remodeled muscular arteries show increased smooth muscle mass, resulting in artery wall thickening, lumen narrowing and vasoreactivity enhancement, with subsequent pulmonary vascular resistance augmentation. In mouse models of asthma, the increase in pulmonary arteries smooth muscle (PASM) mass also persists during short-term disease remission [12, 13].

Because hypoxemia and chronic airway inflammation also occur in severe equine asthma, they may induce remodeling of small pulmonary arteries. If present, pulmonary artery remodeling could then contribute to both pulmonary hypertension onset during clinical exacerbation of the disease and persistence of higher pulmonary artery pressure values during the disease remission. We therefore postulated that pulmonary vascular remodeling occurs in severe equine asthma and is associated with increased PASM mass and vascular lumen narrowing. According to our hypothesis, the present study had the following objectives: to 1) determine the presence and anatomical location of pulmonary artery remodeling in severe equine asthma; 2) assess whether differences are detectable between remission and exacerbation; 3) investigate the remodeling pattern and whether an increased PASM mass is present and; 4) assess remodeling reversibility after long-term asthma treatments.

## Materials and methods

### Study design

This study was part of a larger project aiming to assess lung tissue remodeling and its reversibility in severe equine asthma. The present study focused on remodeling of pulmonary vasculature and particularly of muscular pulmonary arteries, namely small ramifications of the pulmonary arterial trunk that appear to be more prone to remodeling in previous studies on rodent models [17, 18]. A blinded histomorphometric assessment was conducted on lung samples retrieved from the BTRE equine respiratory tissue biobank (http://www.btre.ca) in three different phases (study 1, 2 and 3).

Study 1 consisted of a preliminary study performed on a subset of banked samples selected from a previous cadaveric study [21]. Study 1 aimed to determine the presence of pulmonary artery remodeling and its anatomical location within different lung regions, as well as to assess differences between remission and exacerbation states. The lung samples were collected *post-mortem*, respectively from 6 asthmatic horses in exacerbation (antigen exposure), 6 asthmatic horses in remission (short-term antigen avoidance) and 6 age-matched controls. Samples belonged to different regions of the lung and named cranio-caudally from “A” to “D” as previously described [21]. Sample “E” was collected from the peripheral caudodorsal part of the main lung lobe and corresponds to the specific biopsy site during thoracoscopy. The wall area of muscular pulmonary arteries was measured by histomorphometry, as an estimation of the degree of artery wall thickening. Measurements were compared between asthmatic horses and controls in different lung regions. A comparison between asthmatic horses in remission and exacerbation was also performed.

Study 2 was performed on banked samples from a previous *in vivo* case-control study [22]. Study 2 aimed to confirm *post-mortem* results and to determine the presence of PASM mass involvement in the remodeling. All samples belonged to peripheral lung biopsies collected via thoracoscopy from the caudodorsal lung of 6 asthmatic horses in remission and 5 age-matched controls, as previously described [23]. The pulmonary artery wall area was measured, and the intimal and medial areas were evaluated, to assess presence of specific intimal and/or medial thickening. The PASM mass and the extracellular matrix (ECM) were quantified on sections immunostained for smooth muscle specific α-actin (α-SMA). Measurements were compared between asthmatic horses and controls.

Study 3 was performed on banked samples from an *in-vivo* prospective randomized clinical trial involving 11 asthmatic horses randomly divided into 2 groups and treated for 12 months with two different protocols [24]. Study 3 aimed to assess reversibility of wall and PASM mass changes after long-term asthma treatment. Details concerning the animals used, treatment protocols and sampling procedures are reported elsewhere [24]. Briefly, treatment protocols included antigen avoidance strategy alone for the whole experimental period (samples from 5 horses) or inhaled corticosteroid (fluticasone) alone for the first 6 months, combined with antigen avoidance for the remaining 6 months (samples from 6 horses). The pulmonary artery wall area and PASM mass were quantified by histomorphometry and compared in each treatment group between samples collected at baseline (before treatment, during disease exacerbation) and samples collected at follow-up (end of the treatment period).

### Sample selection

Samples were selected from the caseload available in the BTRE equine respiratory tissue biobank. To be included in the biobank, all the asthmatic horses were required to have a well documented 3–10-year history of disease. The criteria to define severe asthma included presence of recurrent clinical exacerbation of airway obstruction (expiratory dyspnea with abdominal effort and nasal flaring); presence of neutrophilic airway inflammation (BALF neutrophil % > 25% during exacerbation) and/or presence of documented airflow obstruction at lung function testing during exacerbation (transpulmonary pressure, Ppl > 15 cmH_2_0; lung resistance, RL > 1 cmH_2_0/L/s, lung elastance, EL > 1cmH_2_0/L). Clinical remission of the disease was experimentally obtained by keeping asthmatic horses on a low antigenic exposure (pasture or hay alternatives) for 2–4 months before recruitment for the study. Clinical exacerbation was experimentally induced by stabling and hay feeding. Lung function and BALF cytology were assessed as previously described [2, 25]. Control horses were free of respiratory diseases and were age-matched to asthmatic horses. The horses' age, sex, breed, results of lung function testing and BALF neutrophil % for each study are included in the S1 File.

For the post-mortem study, specimens were collected within 2 hours from euthanasia, randomly from the right or left lung. For every horse, one sample of 6–8 cm ^3^ was collected from each one of the following sites: sample “A” was collected from the lung apex; sample “B” was collected at the emergence of the main bronchus; sample “C” was collected from the center of the main lung lobe; sample “D” was collected from the caudodorsal part of the main lung lobe; sample “E” was collected from the peripheral caudodorsal part of the main lung lobe and corresponds to the specific biopsy site during thoracoscopy. Detailed representation of the sites of collection was previously reported [21]. For in-vivo studies, all the available tissue specimens were peripheral biopsies of 8–12 cm^3^ collected from the caudodorsal lung (region E), via standing thoracoscopy [23]. For each horse included in the study (study 2 and 3) and for each assessment (study 3, pre- and post-treatment), an average number of 3–5 specimens were available in the biobank.

### Sample processing and staining

Lung samples were fixed for 24 hours in 4% formaldehyde, embedded in paraffin, cut, placed on glass slides and stained. In study 1, samples were stained for histology with hematoxylin eosin saffron. In study 2 and 3, samples were stained for histology with modified Movat Russell pentachrome [26]. This staining highlights the internal elastic lamina, allowing differentiation between intimal and medial layers within the artery wall. Samples for study 2 and 3 were also immunostained for α-SMA, as previously described [27].

### Histomorphometry

Two different technical approaches were used to assess histomorphometric parameters on histological stained and immunostained sections, respectively.

**Fig 1:**
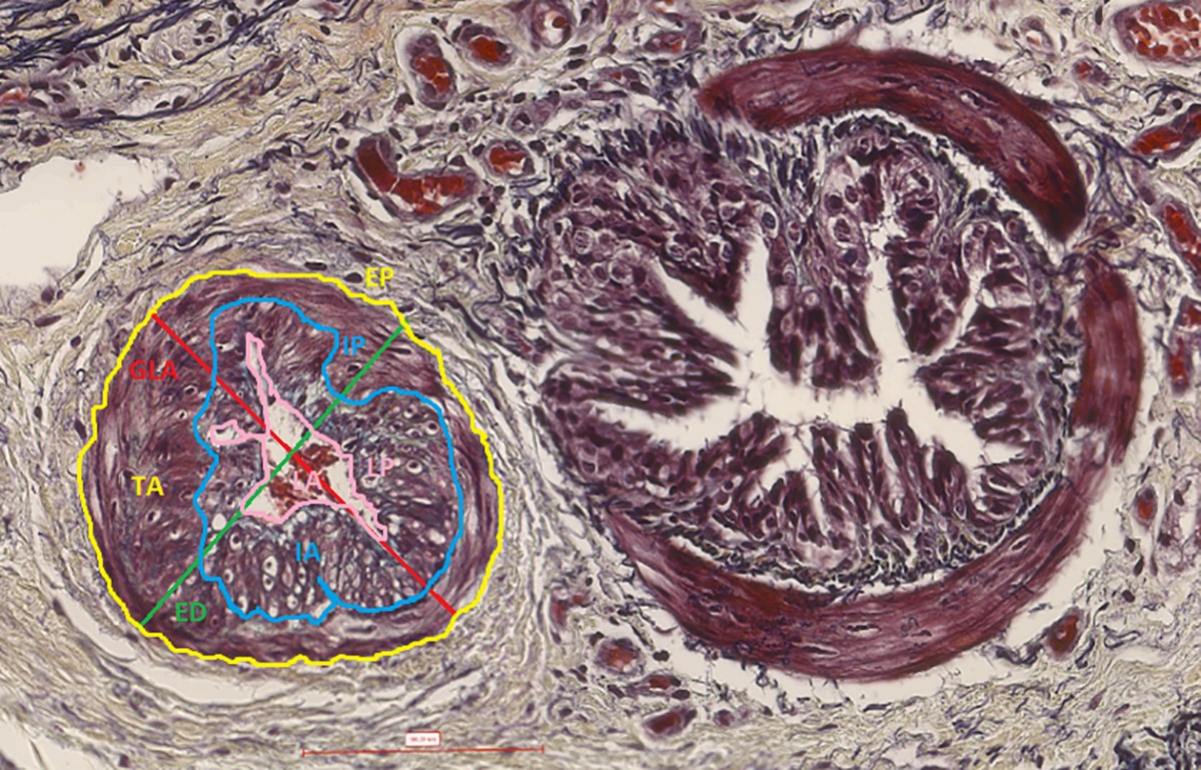
Histomorphometric 1D and 2D measured parameters. Muscular pulmonary artery and annexed bronchus (histological section, 40X, Movat Russel pentachrome). GLA (red outline) = great longitudinal axis; ED (green outline) = external diameter; EP (yellow outline) = external perimeter; IP* (blue outline) = internal perimeter; LP (pink outline) = lumen perimeter; TA (within yellow outline) = total area; IA* (within blue outline) = internal area; LA (within pink outline) = *lumen* area.

*Post-mortem* and *in viv*o histological sections were scanned and digitalized at 40X magnification as TIFF images with PanOptiq^TM^ software version 1.4.3 (ViewsIQ, Vancouver, Canada); morphometric measurements were collected on TIFF images using ImageJ software version 1.4g [28]. This approach allowed estimating the area occupied by the intima and media layers of the arterial wall delimited by elastic fibers (external and internal arterial elastic laminas), as previously described [29]. All the arteries with clear distinction between the wall and the *lumen* were evaluated. Both linear (1D) and bidimensional (2D) parameters were measured or derived, as an adaptation of what was previously reported for human pulmonary vascular histomorphometry [29–31]. Definition and collection methods of 1D and 2D “measured parameters” are summarized in Table 1 and Fig 1. “Derived parameters” were calculated from “measured parameters”, according to definitions summarized in Table 2 (Office Excel^R^ software v. 2016; Microsoft Corporation, Redmond, USA). In horses, muscular pulmonary arteries have a minimal external diameter (ED) of 40 μm therefore, arteries with lower ED length were excluded [32]. Narrowing index and GLA (great longitudinal axis)/ED *ratio* were used to assess differences due to histological processing, artery collapse and cut sections respectively. Longitudinal cut sections of vessels (GLA/ED *ratio* > 3) were excluded, as previously suggested [29]. The internal perimeter (IP) and the internal area (IA) were measured on Movat Russell pentachrome stained sections (study 2), only if the internal elastic lamina appeared well defined in the whole artery. The intimal area and the medial area were obtained only if IA measurement was available. To allow comparison among data of different sized-artery sections, these measurements were also expressed as percentages of the total area (wall area %, intimal area % and medial area %, see Table 2).

**Table 1:** Histomorphometric linear (1D) and bidimensional (2D) measured parameters.

**Linear (1D) measured parameters (μm)**	
External artery diameter (ED)	Widest distance between external elastic laminas, perpendicular to the greatest longitudinal axis
Great longitudinal axis (GLA)	Longest distance between external elastic laminas
External perimeter (EP)	Outline of the external elastic lamina
Internal perimeter (IP)^a^	Outline of the internal elastic lamina
*Lumen* perimeter (LP)	Outline of the inner aspect of the intima
**Bidimensional (2D) measured parameters (μm**^**2**^**)**	
Total area (TA)	Area encompassed by the external perimeter
Internal area (IA)^a^	Area encompassed by the internal perimeter
*Lumen* area (LA)	Area encompassed by the lumen perimeter

^a^Assessed only on sections stained with Movat Russell pentachrome (study 2).

**Table 2:** Histomorphometric bidimensional and immunostained derived parameters.

Wall area (WA) (μm^2^)	Area included between the external perimeter and the lumen perimeter, calculated as difference between total measured area and lumen area
Wall area %	Wall area expressed as a percentage of the total measured area, calculated as follows: (WA/TA) *100
Medial area (MA) (μm^2^) ^a^	Area included between the external perimeter and the internal perimeter, calculated as difference between total measured area and internal area
Medial area %	Medial area expressed as a percentage of the total measured area, calculated as follows: (MA/TA) *100
Intimal area (ImA) (μm^2^) ^a^	Area included between the internal perimeter and the lumen perimeter, calculated as difference between internal area and lumen area
Intimal area %	Intimal area expressed as a percentage of the total measured area, calculated as follows: (ImA/TA) *100
GLA/ED *ratio*	Ratio between greatest longitudinal axis (GLA) and external artery diameter (ED)
Narrowing index	Index of artery collapse estimated as the *ratio* between the total measured area (TA) and the theoretical area. The theoretical area represents the area of the fully distended artery obtained from a theoretical diameter calculated as follows: external perimeter (EP)/π
α-SMA positive wall area % (α-SMA + area %, PASM mass)	Wall area positive for the α-SMA immunostaining, expressed as a percentage of the total measured area of the vessel, calculated as follows: (∑p_α-SMA+_ /∑p_vessel_)*100
α-SMA negative wall area % (α-SMA—area %, ECM mass)	Wall area negative for the α-SMA immunostaining, expressed as a percentage of the total measured area of the vessel, calculated as follows: (∑p_α-SMA-_ /∑p_vessel_)*100

^a^Assessed only on sections stained with Movat Russell pentachrome (study 2).

**Fig 2:**
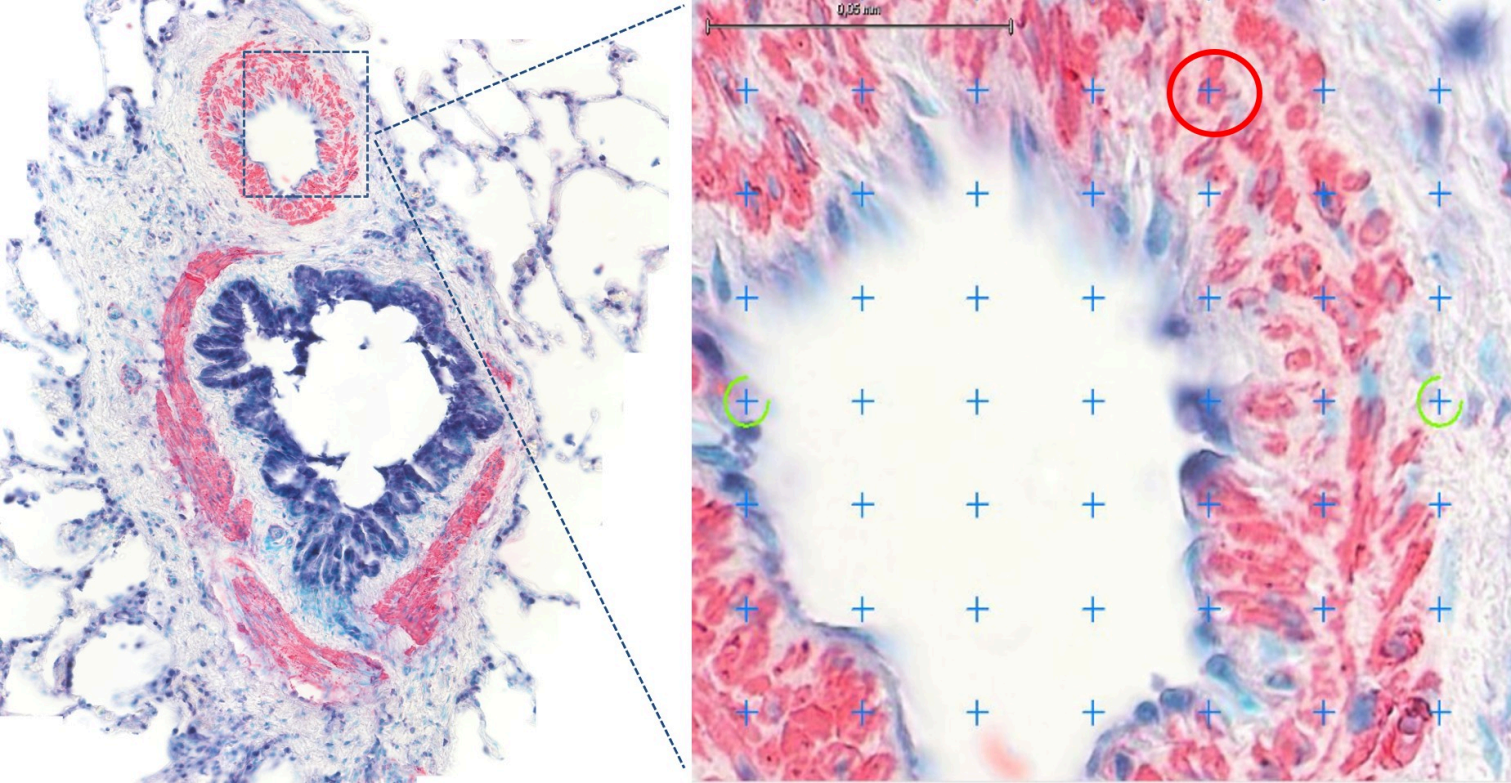
Immunostaining for α-smooth muscle actin (α-SMA) and point counting analysis. Pulmonary arteries smooth muscle (PASM) stained pink-red with immunostaining for α-SMA, while extracellular matrix (ECM) did not stain. An example of region of interest (ROI) analysis with the point counting technique is provided. A grid of 576 crosses/screen was applied. Crosses falling on pink-red artery wall points (red circle) were counted as α-SMA+ (representative of PASM). Crosses falling on uncolored artery wall points (yellow circle) were counted as α-SMA- (representative of ECM).

Immunostained muscular pulmonary arteries were scanned at 40X magnification and digitalized as SVS images with PanOptiq^TM^ software version 1.4.3 (ViewsIQ, Vancouver, Canada). Histomorphometry was then performed on the SVS images using the NewCAST^TM^ software version 4.5.1.324 (Visiopharm Integrator System, Hoersholm, Denmark). The total vessel area (μm^2^) and the α-SMA positive and negative wall area (μm^2^) were estimated with a counting point technique. Because it was postulated that vascular smooth muscle increases with artery wall remodeling, this approach allowed estimating specifically the smooth muscle component, independently from its location within the artery wall (intimal and/or medial layer). Immunostaining for α-SMA has been used to quantify vascular smooth muscle mass in rodent models [16, 18]. A similar point-counting technique had been used to estimate airway smooth muscle mass remodeling in severe equine asthma [33]. Briefly, on each uploaded SVS digital image, every visible pulmonary artery section was delimited as a region of interest (ROI), using the “ROI drawing” function. The parameters counted were the sum of the points falling on vessel lumen and wall areas (∑p_vessel_), the sum of the points falling on α-SMA positive wall area (∑p_α-SMA+_) and the sum of the points falling on α-SMA negative wall area (∑p_α-SMA-_). A grid of 576 crosses per screen was applied providing a total count of *at least* 400 points for every counted parameter in each horse ensuring reliable individual estimation according to the current stereology guidelines [34]. The total vessel area, the α-SMA positive wall area and the α-SMA negative wall area were estimated by multiplying the ∑p_vessel_, ∑p_α-SMA+_ or ∑p_α-SMA-_ respectively per the area subtended to a single point (117,22 μm^2^). The total vessel area reflects the overall area occupied by the artery section (both *lumen* and wall area), while the α-SMA positive wall area and the α-SMA negative area reflect respectively the PASM and ECM mass within the artery wall (Fig 2). Both α-SMA positive and negative wall areas were then expressed as a percentage of the total vessel area (α-SMA + area %; α-SMA—area %), to allow comparison among data of different sized-artery sections (see Table 2).

### Statistical analysis

Previous studies have demonstrated statistically significant differences in lung tissue remodeling and its reversibility between asthmatic horses and controls using samples of 5–6 horses in each group [22, 27]. Therefore, samples from 6 different horses for each group (controls, asthma remission and asthma exacerbation) were included to perform the preliminary study (study 1). Preliminary data obtained in study 1 were used to perform a power analysis, confirming that tissue samples from 5 horses per group will be sufficient to demonstrate statistically significant differences between controls and asthmatic horses in remission (α = 0.05; *p* = 0.90). Because groups were small, normality was assessed by visual inspection of the data. All comparisons were performed using *t* Student test with Welch’s correction (study 1 and 2) and paired *t* test (study 3). One-tailed tests were used to increase power in comparing groups for the wall area%, intimal area%, medial area%, α-SMA+ and—area %, because according to the hypothesis, significant changes were expected only in one direction (increase in asthmatic samples for study 1 and 2, decrease at follow up for study 3). Statistical tests were performed with GraphPad Prism^TM^ software v. 6 (GraphPad software Inc., California, USA). The level of statistical significance was set at p < 0.05.

## Results

### Pulmonary arteries

Table 3 summarizes the average numbers of histological and immunostained arteries assessed in each study. In study 1, a total number of 222, 272 and 230 sections of different arteries were assessed respectively in the controls (median/horse of 37 arteries, range 19–51), asthmatic horses in remission (median/horse of 43 arteries, range 31–66) and exacerbation (median/horse of 33 arteries, range 27–68). In study 2, a total number of 93 histological (median/horse of 15 arteries, range 10–32) and 111 immunostained (median/horse of 22 arteries, range 14–32) sections of different arteries were assessed for the control group. For the asthmatic group, total histological sections were 116 (median/horse of 17 arteries, range 7–44) and total immunostained sections were 114 (median/horse of 17 arteries, range 8–33). In study 3, for the group that received steroids, the total number of histological sections evaluated was 46 (median/horse of 8 arteries, range 4–12) at baseline and 123 (median/horse of 18 arteries, range 13–36) at follow up; the total number of immunostained sections evaluated was 74 (median/horse of 12 arteries, range 4–23) at baseline and 97 (median/horse of 14 arteries, range 6–32) at follow up. For the group that was treated only with strict antigen avoidance, the total number of histological sections evaluated was 58 (median/horse of 7 arteries, range 2–26) at baseline and 126 (median/horse of 26 arteries, range 4–42) at follow up. The assessment of the immunostained sections in this group was performed on 4 horses, because one horse had only two arteries sections measurable at the baseline. The total number of immunostained sections was 73 (median/horse of 15 arteries, range 11–32) at baseline and 51 (median/horse of 13 arteries, range 6–19) at follow up.

**Table 3:** Total number, median and ranges of assessed histological and immunostained pulmonary arteries.

Study /Group	Horses (n)	Histology					Immunostaining				
		Arteries (total)		Median/ horse		Range	Arteries (total)		Median/ horse		Range
**Study 1**											
Controls	6	222	37		19–51		-	-		-	
Asthma	12	502	36		27–68		**-**	**-**		**-**	
• Remission	6	272	43		31–66						
• Exacerbation	6	230	33		27–68						
**Study 2**											
Controls	5	93	15		10–32		111	22		14–32	
Asthma	6	116	17		7–44		114	17		8–33	
**Study 3**											
Antigen avoidance											
• Baseline	5 (4)^a^	58	7		2–26		73^a^	15^a^		11–32^a^	
• Follow up	5 (4)^a^	126	26		4–42		51^a^	13^a^		6–19^a^	
Corticosteroids											
• Baseline	6	46	8		4–12		74	12		4–23	
• Follow up	6	123	18		13–36		97	14		6–32	

^a^In study 3, one horse of the antigen avoidance group was not considered for the statistical analysis of the pulmonary arteries smooth muscle mass quantification because at the baseline only one immunostained artery was measurable.

### Study 1

**Fig 3:**
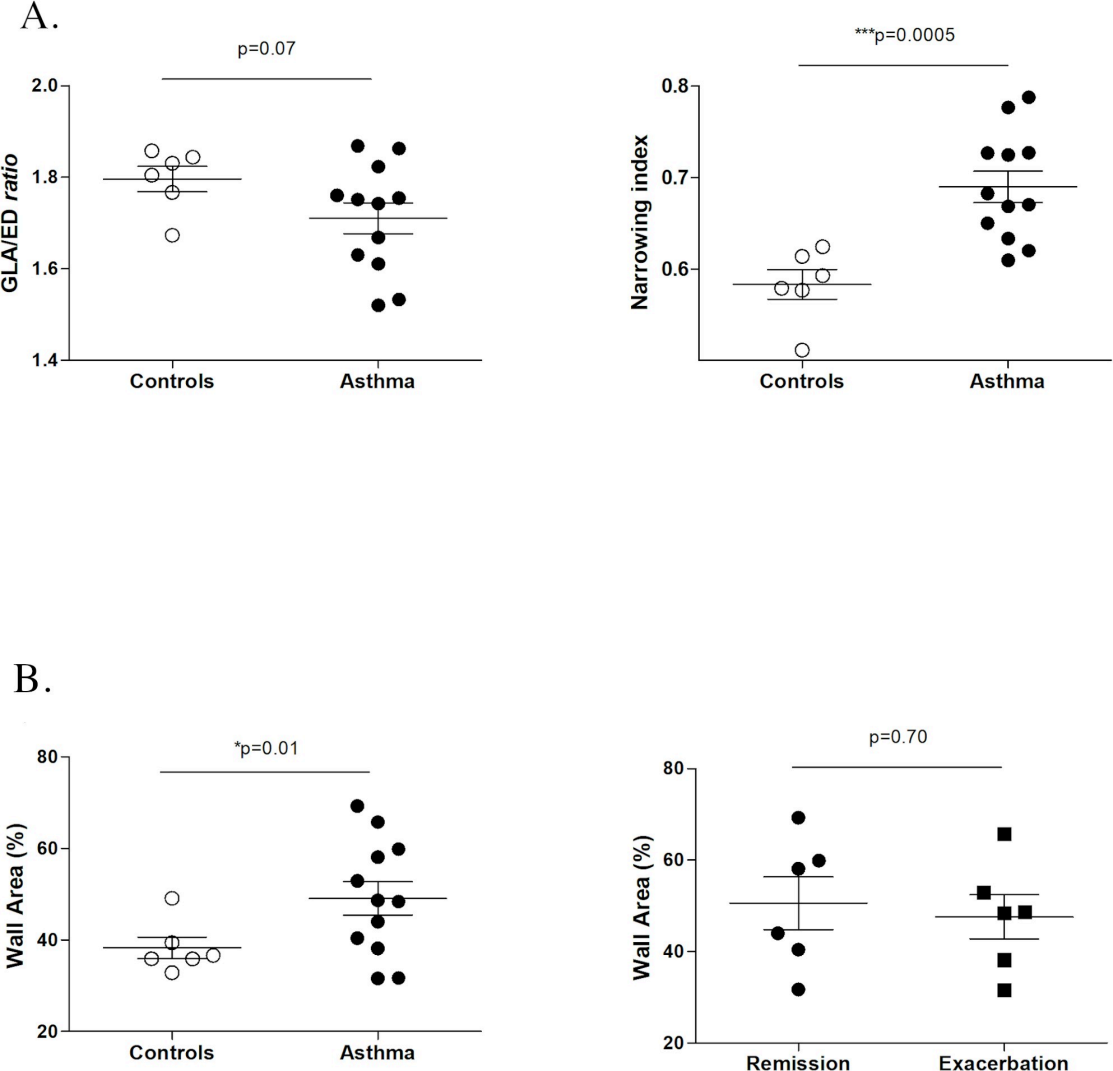
Summary of study 1 results. Comparison between asthmatic horses and controls of GLA (great longitudinal axis)/ED (external diameter) *ratio* (3a), narrowing index (3a) and wall area % (3b).

**Fig 4:**
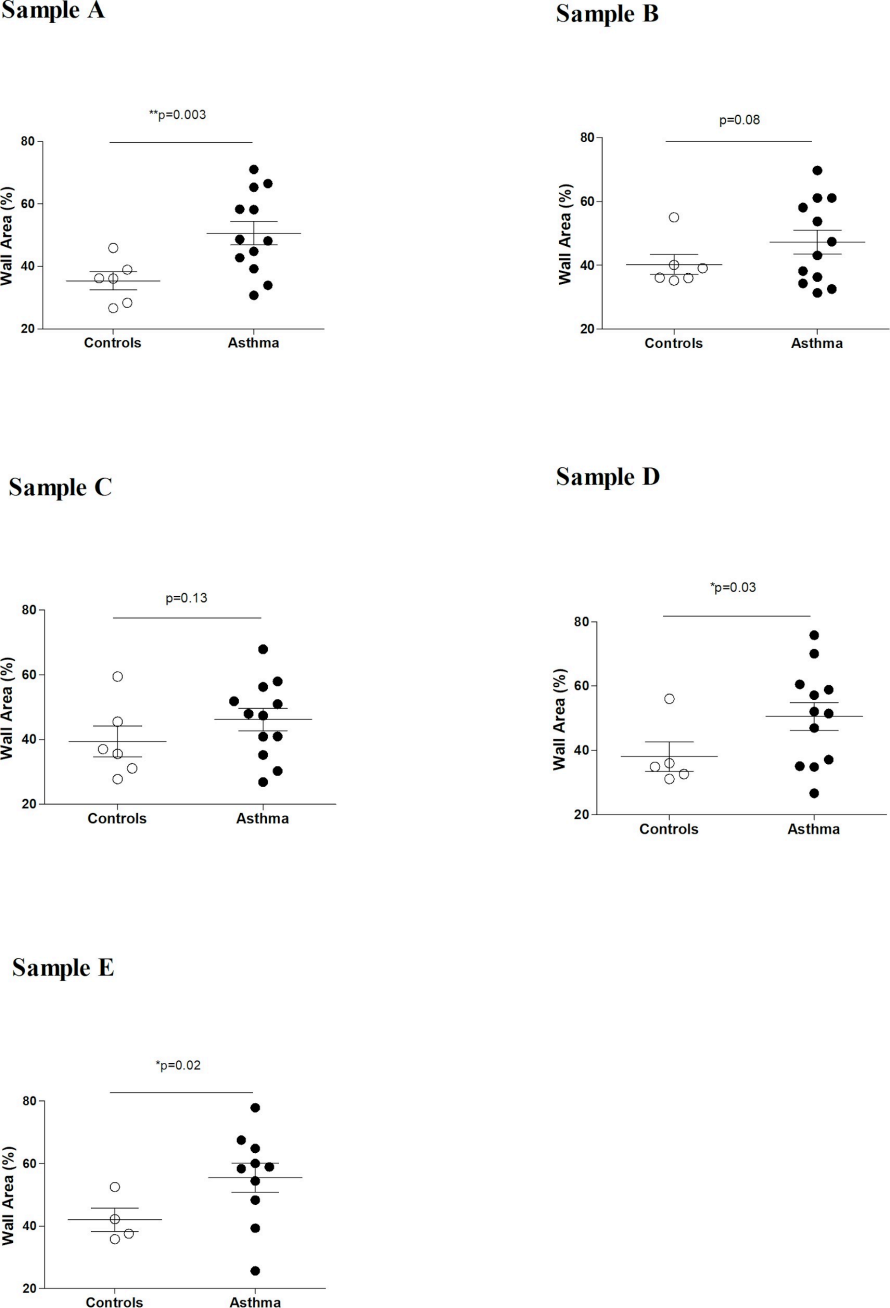
Summary of study 1 results: wall area % in different lung regions (samples “A”-“E”). Significant wall thickening (increased wall area %) was detected in sample “A”, sample “D” and sample “E”. As previously described [21], sample “A” was collected from the lung apex, sample “B” at the emergence of the main bronchus, sample “C” from the center of the main lung lobe, sample “D” from the caudodorsal part of the main lung lobe and sample “E” was collected from the peripheral caudodorsal part of the main lung lobe and corresponds to the specific biopsy site during thoracoscopy.

**Fig 5:**
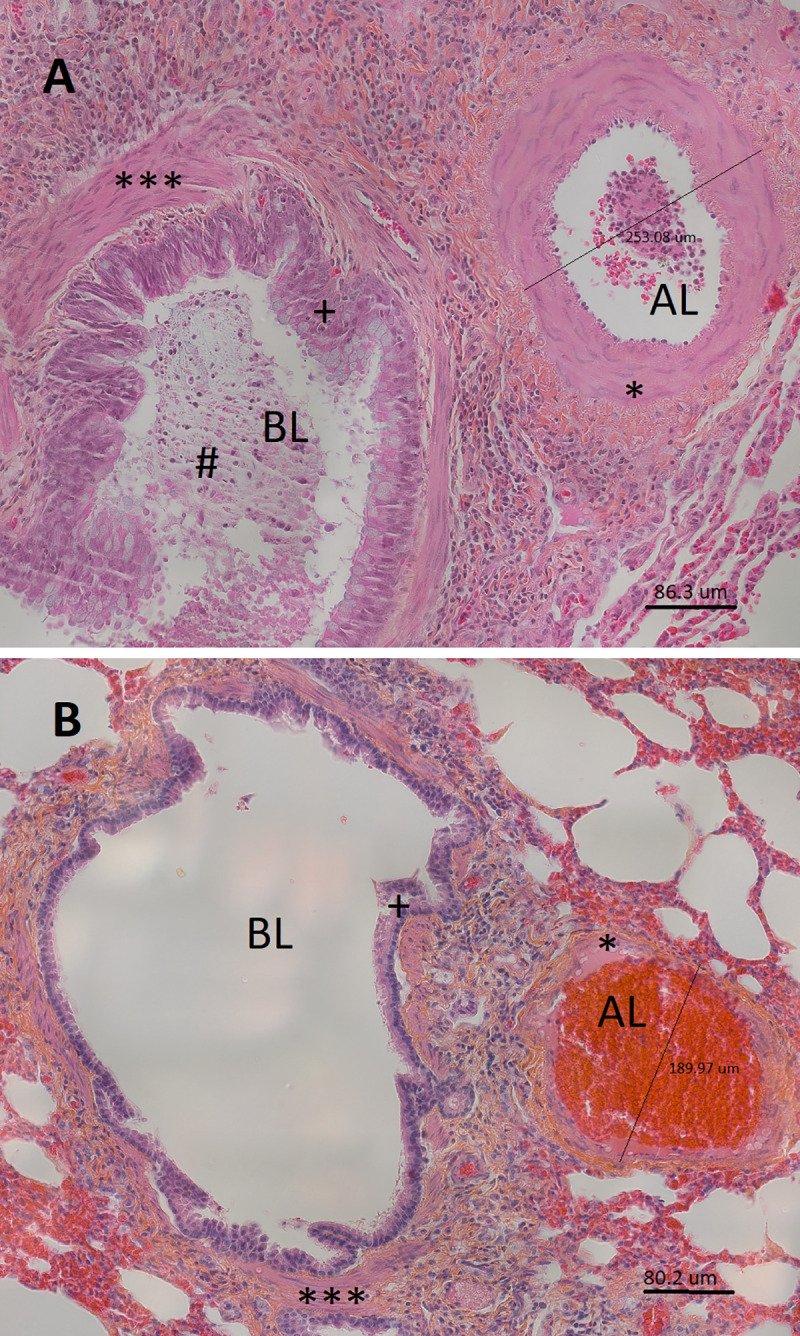
Muscular pulmonary artery and annexed bronchus of an asthmatic horse in exacerbation (A) and of a control horse (B) in study 1 (histological sections, 40X, hematoxylin eosin saffron). The bronchus lumen (BL) and artery lumen (AL) are identified. The measurement of the artery external diameter is also reported. In these micrographs, thickening of the muscular pulmonary artery wall (*) is visually detectable in the asthmatic horse (A), compared to a control animal (B). Notably, asthma-related airway remodeling is also detectable in the micrograph from the asthmatic horse (A), with mucus accumulation in the airway lumen (#), epithelial thickening (+) and increased airway smooth muscle mass (***), compared to a control (B).

Figs 3 and 4 summarize the results from study 1. Because the GLA/ED ratio did not differ between asthmatic and control horses (p = 0.07), the cutting angle was assumed to have minimal, if any, impact in the morphometric assessment. In asthmatic horses, pulmonary arteries had significantly greater narrowing index (p = 0.0005), i.e. the total measured area was close to the area of the theoretical fully distended artery. That means that pulmonary arteries in asthmatic horses were significantly less collapsed. Furthermore, asthmatic horses had thickened (significantly greater wall area %, p = 0.01) pulmonary arteries, compared to controls (Fig 5). However, no significant differences in the wall area % were detected between asthmatic horses in remission and asthmatic horses in exacerbation (p = 0.70). That means that short term remission did not affect the pulmonary artery wall thickening. Pulmonary artery wall thickening (greater wall area %) was detected in the apex (sample A, p = 0.03) and caudodorsal lung fields (sample D, p = 0.03), but not in the main lobe (sample B and C, p = 0.08 and p = 0.13, respectively). Significant increase in wall area % was also detected in the specific region sampled via thoracoscopy (sample E, p = 0.02).

### Study 2

**Fig 6:**
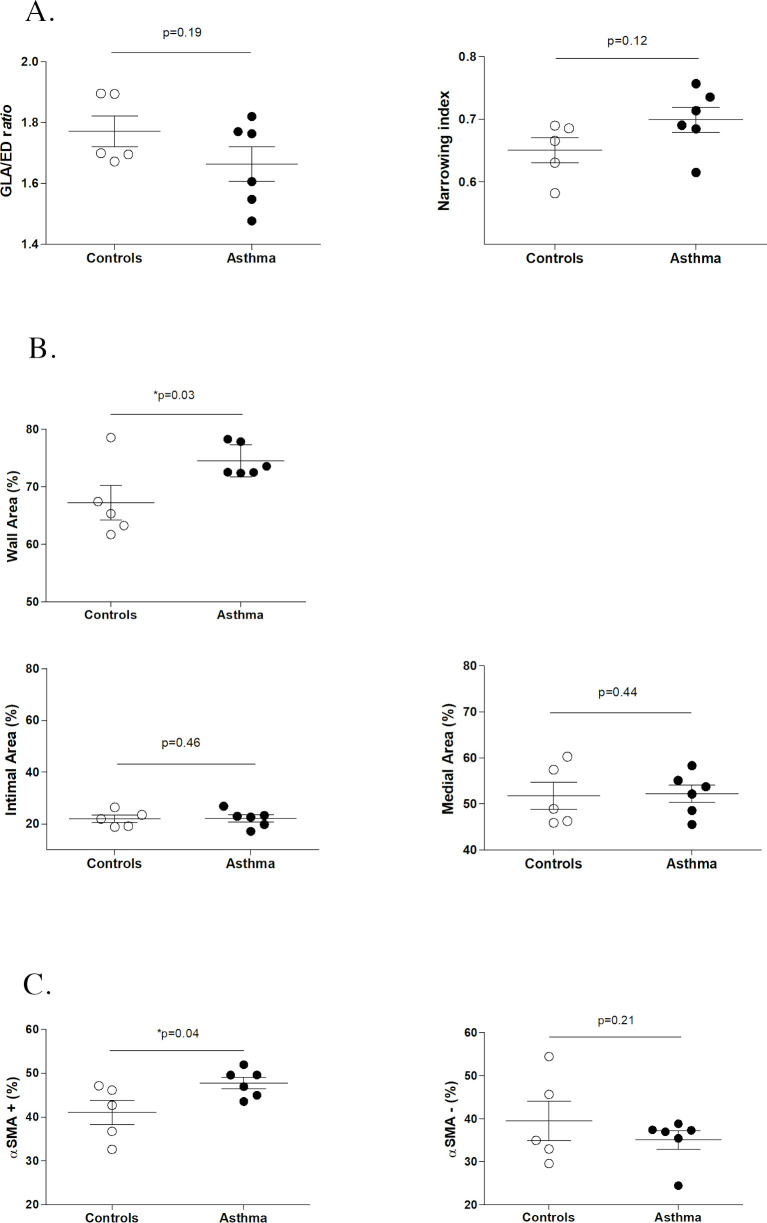
Summary of study 2 results. Comparison between asthmatic horses and controls of GLA (great longitudinal axis)/ED (external diameter) *ratio* (5a), narrowing index (5a), wall area % (5b), α-SMA+ (α-smooth muscle actin positive) area % (5c) and α-SMA- (α-smooth muscle actin negative) area % (5c).

**Fig 7:**
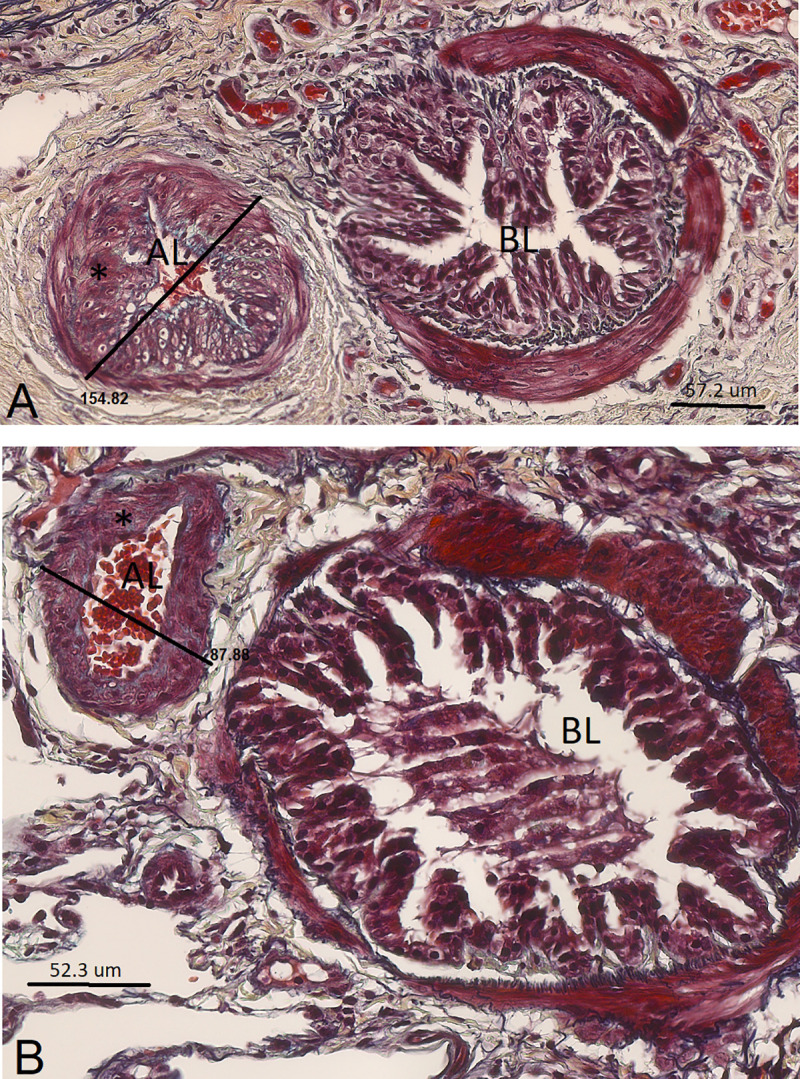
Muscular pulmonary artery and annexed bronchus of an asthmatic horse in remission (A) and of a control horse (B) in study 2 (histological sections, 40X, Movat Russel pentachrome). The bronchus lumen (BL) and artery lumen (AL) are identified. The measurement of the artery external diameter is also reported. In these micrographs, thickening of the muscular pulmonary artery wall (*) is visually detectable in the asthmatic horse (A), compared to a control animal (B).

**Fig 8:**
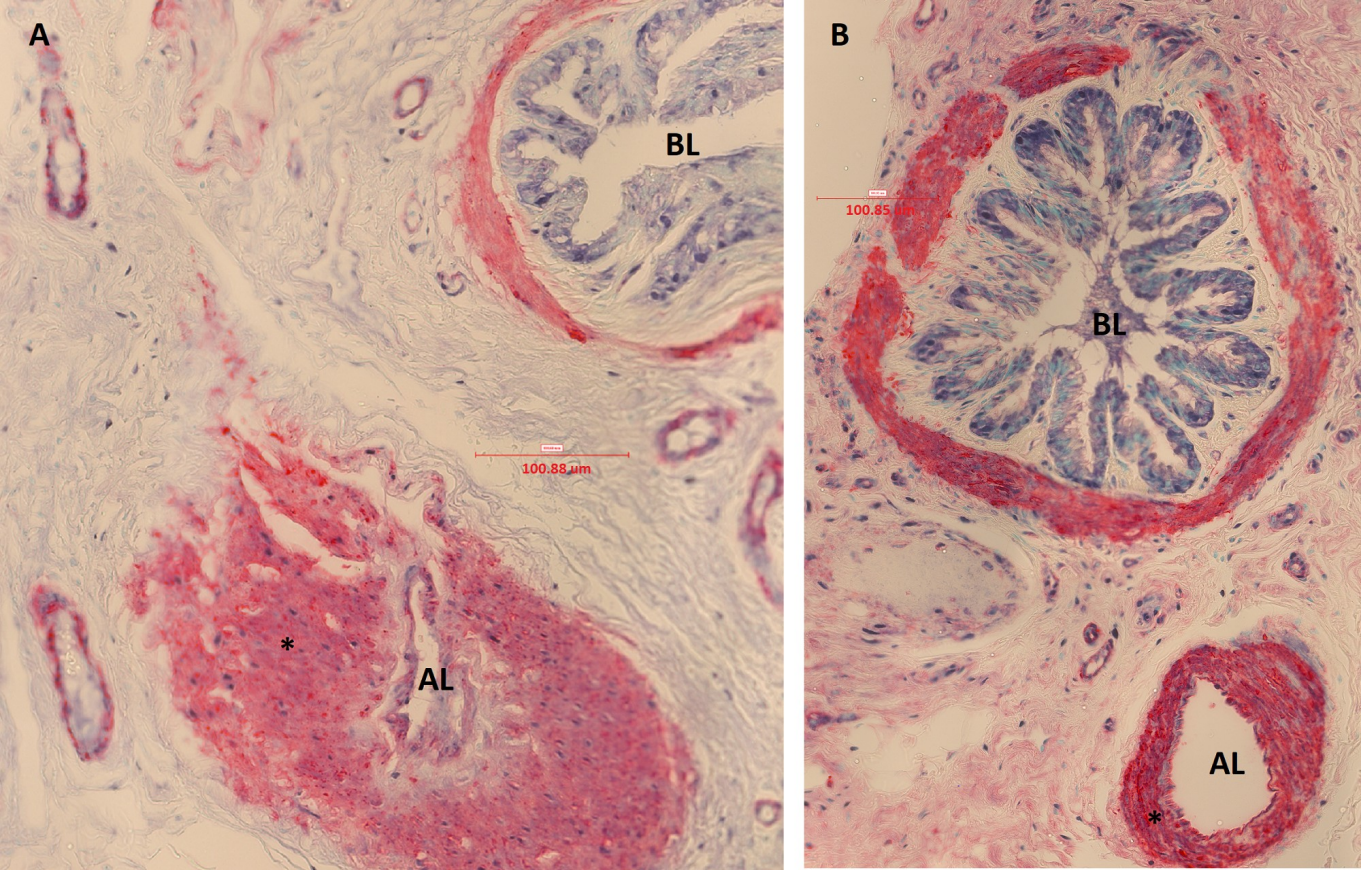
Muscular pulmonary artery and annexed bronchus of an asthmatic horse in remission (A) and of a control horse (B) in study 2 (immunostained sections, 40X, α-smooth muscle actin). The bronchus lumen (BL) and artery lumen (AL) are identified. In these micrographs, significant increase in vascular smooth muscle (*) of the muscular pulmonary artery is visually detectable in the asthmatic horse (A), compared to a control (B).

Fig 6 summarizes results from study 2. Asthmatic horses and controls did not differ in respect to the cut section (GLA/ED ratio, p = 0.19), so similarly to study 1, the cutting angle was assumed to have no impact in the morphometric assessment. Conversely to study 1, there was no significant difference between asthmatic horses and controls in the narrowing index (p = 0.12). In both groups, the narrowing index was in the higher range (between 0.5 and 1), meaning that the total measured area was close to the area of the theoretical fully distended artery (low degree of vascular collapse). Similarly to study 1, asthmatic horses showed significant pulmonary arteries wall thickening (greater wall area %) compared to controls (p = 0.03, Fig 7). However, the wall thickening was not associated with an increase in % of medial area or % of intimal area in asthmatic horses compared to controls (p = 0.46 and p = 0.44, respectively). Immunostaining revealed that in asthmatic horses, the increase in % of wall area corresponded to a significant increase in the % of PASM mass (α-SMA+ area %, p = 0.04) but not in ECM (α-SMA- area %, p = 0.21) (Fig 8).

### Study 3

**Fig 9:**
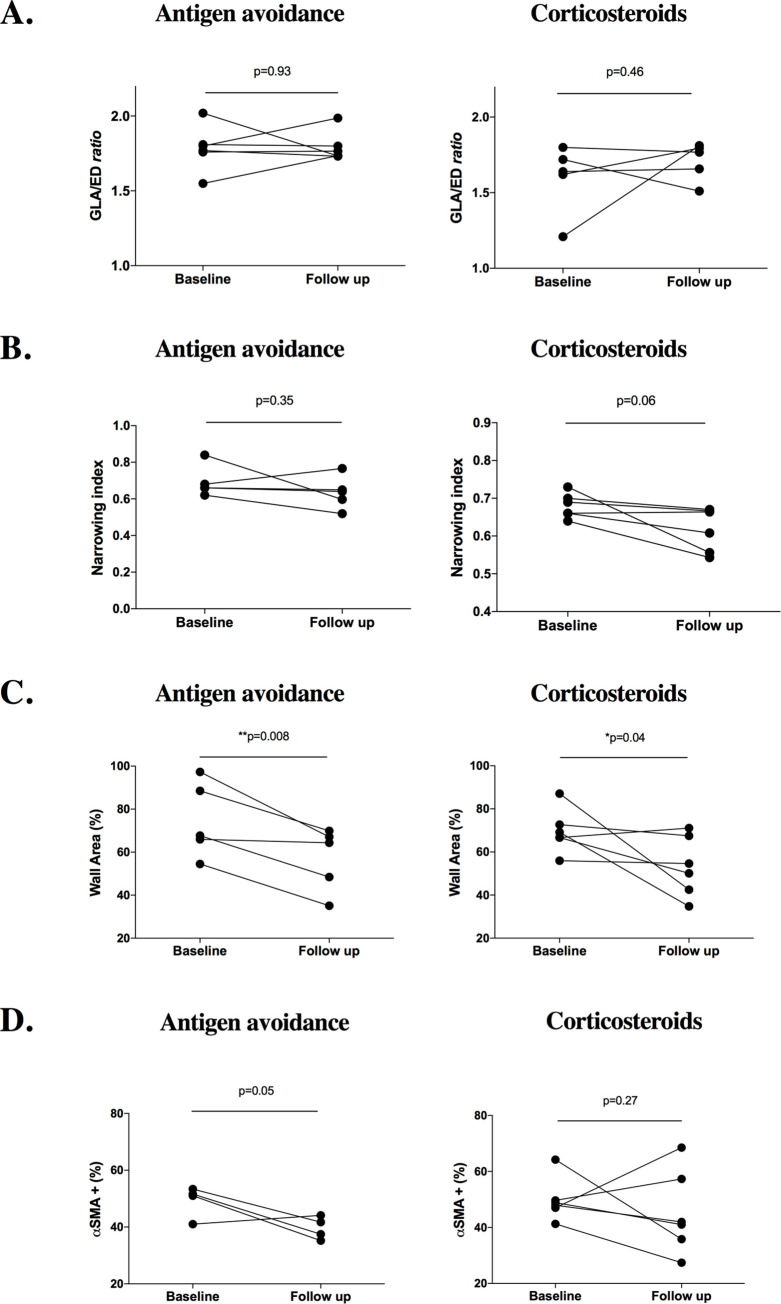
Summary of study 3 results. Comparison between baseline and follow up in both groups of GLA (great longitudinal axis)/ED (external diameter) *ratio* (6a), narrowing index (6b), wall area % (6c), α-SMA+ (α-smooth muscle actin positive) area % (6d). A significant reduction of the wall thickening (wall area%) was observed at the follow-up in both groups, although reduction was more uniform in the antigen avoidance group.

Fig 9 summarizes results from study 3. In both treatment groups, no differences in respect to histologic cut sections (GLA/ED ratio) were found between baseline and follow up (p = 0.93 and p = 0.46), so similarly to study 1 and 2, the cutting angle was assumed to have little or no impact on the morphometric assessment. Also, the narrowing index not significantly different between baseline and follow-up in both groups (p = 0.35 and p = 0.06). Long-term treatment induced significant reduction of the wall area (p = 0.008 and p = 0.04) in both groups. However, visually the antigen avoidance strategy allowed more consistent reduction of the wall area (all the horses of the group) while treatment with steroids was not effective in 2 of the 6 horses included in the treatment group. In addition, only the antigen avoidance strategy was associated with a visual trend in PASM % reduction, although not significant (p = 0.05). Assessment of the PASM% reduction in the antigen avoidance group might have been underpowered because one horse was not included in the statistical analysis (see Table 3). Treatment with steroids did not show any significant effect on the PASM% (p = 0.27).

## Discussion

The present study revealed significant pulmonary artery wall thickening (increase in the pulmonary artery wall area) in asthmatic horses compared to controls. Remodeling involved the lung apex and the caudodorsal lung fields, including the specific region sampled by *in viv*o thoracoscopy. In asthmatic horses, pulmonary artery wall thickening was due to an increase in PASM mass, but the ECM was unaffected. Remodeling changes (wall area and PASM increase) were uniformly reversible after long-term antigen avoidance (12 months), but not after 2 to 4 months, nor after 12 months of inhaled corticosteroids combined with 6 months of antigen avoidance.

This is the first study that used a quantitative histomorphometric approach to assess pulmonary vascular remodeling in horses. The mean wall area of muscular pulmonary arteries in asthmatic horses was about 7% and 12% greater than that of controls, *in vivo* and *post-mortem* respectively. Increases of similar magnitudes (7–10%) were observed in the lungs of COPD-affected human patients compared to controls [29, 35, 36]. *Post-mortem*, pulmonary arteries of asthmatic horses appeared significantly less collapsed (higher narrowing index) than those of controls. This difference was not observed *in vivo*, with both groups showing similar low degree of collapse. Pulmonary arteries of asthmatic horses may show hypercontractility and impaired *post-mortem* relaxation similarly to what was previously demonstrated in experimental animal models of lung allergic inflammation [37–39]. However, differences in wall area (expression of wall thickening) were not affected by reactivity differences between groups and were similarly detected both *in vivo* and *post-mortem*, suggesting consistent remodeling of pulmonary artery walls in asthmatic horses.

Vascular remodeling affected only the caudodorsal lung fields and the lung apex. Lung blood flow distribution in horses increases in a dorsal and caudal direction [40, 41]. Higher perfusion of caudodorsal lungs may mean higher delivery of inflammatory cells and factors during acute lung inflammation, potentially contributing to selectively located remodeling. Caudodorsal lung fields represented the main site of vascular remodeling also in equine exercise-induced pulmonary hemorrhage (EIPH) [40, 42]. Presence of vascular remodeling in the lung apex was surprising, however. Despite low blood flow, cranioventral small pulmonary arteries appeared more prone to remodeling also in a previous study [43]. The causes are likely due to regional differences in vessel mechanical reactions in response to yet unidentified stimuli that may also be evoked in severe equine asthma. As a hypothesis, and similarly to what is described in human COPD, endothelial dysfunction may occur in severe equine asthma. [19, 35, 36, 44]. In human COPD, this results in impaired production of vasodilators, particularly nitric oxide, which has a significant vasodilatory role in the cranioventral lung. Of note, pulmonary artery remodeling was demonstrated *post-mortem* in the same region sampled by *in vivo* thoracoscopy (sample E). This technique appeared adequate to prospectively investigate *in vivo* vascular remodeling. However, independent of group, pulmonary arteries were 25–30% thicker when lung samples were collected *in vivo* than *post-mortem*. Methodological factors may have been the cause of the difference. In humans, minimally invasive techniques applying high traction force to harvest systemic vessels impair endothelium-dependent relaxation, without affecting contractile response [45].

Presence of vascular remodeling in asthmatic horses was supported by the significant increase in the PASM, in agreement with what has been reported in other chronic spontaneous lung disorders, such as human COPD or equine EIPH, and in rodent asthma models [16–18, 42, 46–48]. In EIPH, a common condition in high intensity performing horses, pulmonary arteries remodeling was also characterized by artery wall thickening with medial smooth muscle hypertrophy, intimal hyperplasia, elastic lamina inconsistency and lack of organization [42]. Severe asthmatic horses did not show specific thickening of the intimal and/or medial layer, however. Pulmonary artery remodeling could have been irregular with total increase of wall PASM mass that was not consistent within a single layer. Absence of specific intimal/medial thickening could also depend on the presence of some degree of elastic lamina inconsistency that prevented intimal and medial area differentiation in all arteries and reduced power of the histomorphometric assessment. Remodeling itself may have caused loss of internal elastic lamina integrity and staining definition, as occurring in equine EIPH.

Persistent hypoxic pulmonary vasoconstriction due to chronic hypoxia resulted in PASM “work” hypertrophy in experimental animal models and a similar pathophysiology could explain the PASM increase of severe asthmatic horses [8, 9, 49]. Airway inflammation may also have contributed. In rodent models, allergic airway inflammation increased PASM mass and cell turnover with similar time course and magnitude to airway smooth muscle remodeling, both being not reversible after one month of antigen avoidance [16, 17]. An increase in airway smooth muscle mass was also present in severe asthmatic horses [22, 27]. Short-term antigen avoidance significantly improved lung function and airway inflammation in asthmatic horses, while smooth muscle mass was not reduced in muscular pulmonary arteries in the present study, nor in small peripheral airways in a previous work [22]. As the PASM cell turnover was not investigated, whether the increase in PASM mass was due to cell proliferation, hyperplasia or hypertrophy was not determined.

Experimentally induced hypoxemia increases mean pulmonary artery pressure both in normal and in asthmatic horses; however, only asthmatic horses develop pulmonary hypertension [11]. In horses with severe asthma, the increased pulmonary artery PASM mass could contribute to pulmonary hypertension during disease exacerbation, by enhancing hypoxic pulmonary vasoconstriction. Furthermore, even when hypoxemia is resolved (disease remission), mean pulmonary artery pressure values are significantly higher in asthmatic horses than controls, although within physiological ranges in both groups. The persistence of PASM mass increase and related artery wall thickening during disease remission may explain why asthmatic horses have persistent higher mean pulmonary artery pressure [11]. In the present study, pulmonary artery remodeling did not affect the amount of vascular ECM. In rodents, increased collagen was detected during sustained exacerbation mainly in the perivascular layer that was not examined in the present study [16, 18]. Perivascular changes and especially ECM deposition merit further investigation in severe equine asthma.

Pulmonary artery remodeling (wall thickening) was reversed by long-term (12 months) antigen avoidance. In horses treated with long-term (12 months) inhaled corticosteroids, remodeling was not reversed, despite 6 months of concomitant antigen avoidance. While inhaled corticosteroids were mainly effective on airway obstruction, long-term antigen avoidance better controlled neutrophilic airway inflammation [24]. This supports a possible role of inflammation in the development of pulmonary artery remodeling, facilitated by the strict anatomic relationship between pulmonary circulation and the airways, as already investigated in acute allergic human asthma [50]. Otherwise, treatment with inhaled corticosteroids could result in vasorelaxation, as expressed by the visually higher degree of collapse. Although this remains to be ascertain, both *in vitro* and experimental *in vivo* studies have shown that corticosteroids may improve the relaxation response of pulmonary arteries by decreasing oxidative stress, up-regulating the expression of nitric oxide synthases and increasing bioavailability of nitric oxide in pulmonary endothelial cells [51, 52].

The present study has several limitations, mainly related to the sampling procedure and the morphometric approaches that were used. Study 1 was performed on samples collected from the whole lung, while studies 2 and 3 were performed only on biopsies obtained from the caudodorsal lungs. Study 1 allowed demonstrating that pulmonary artery remodeling was mainly located in the caudodorsal lung and in the apex, supporting the choice of using peripheral biopsies of the caudodorsal lung for further investigations. The main advantage of using caudodorsal lung peripheral biopsies consists of studying *in-vivo* remodeling evolution and particularly the response to treatment in the same individuals affected with asthma-like lifelong spontaneous disease [1]. This is a unique experimental model for asthma-like disease as other animal models investigating the evolution of remodeling and response to treatment are terminal and performed on relatively short-term experimentally induced models of allergen exposure, not reflecting the lifelong chronicity of the disease [16–18]. However, it must be recognized that limited non-random sampling of the caudo-dorsal lung might not be representative of how the vascular remodeling process evolve and respond to treatment in other parts of the lungs, especially considering the regional differences in perfusion and vasoreactivity that were previously demonstrated in the equine lung [41, 43, 44]. This potentially has an impact on the implications of the present study in clinical practice and could explain why incidence of clinically significant pulmonary hypertension and *cor pulmonale* is only occasional in asthmatic horses, despite the severity and chronicity of the disease.

Another limitation of our study is the absence of randomization of orientation in the sampling procedure that is recommended by stereology guidelines [34]. In the present study, only preferential sections where pulmonary arteries were spatially oriented in a way to be morphologically recognizable were considered, which is not isotropic. The histological stains and especially the immunostaining for the α-smooth muscle actin allow to identify the presence of smooth muscle, however, do not differentiate airways from vascular components. Therefore, the absence of a specific marker for the vascular smooth muscle prevented the use of an isotropic approach. The use of a random tissue orientation techniques of sectioning (such as the isector) would include tangential sections where distinguishing from airways from arteries would be not possible.

Two different histomorphometric approaches were used to address different aspects of the remodeling process. The measurement of areas on the histologically stained section was an efficient technique to assess how the remodeling impacts the arterial wall/lumen ratio. In addition, it allowed investigating whether preferential thickening of the intimal and/or medial layer occurred, as previously also studied in human COPD [46]. On the other hand, the point-counting technique provided an indication of which tissue component (vascular smooth muscle and/or extracellular matrix) was involved in the remodeling process, independently from the location within the arterial wall (intima and/or media layers). Previous studies investigating human COPD demonstrated that significant changes of the histological architecture of the vessel wall occurring with the disease, such as neo-muscularization of the intimal layer [46]. Although data obtained with the two different histomorphometric approaches used are not comparable ones to each other, it is interesting that findings obtained with both techniques were consistent with the presence of the remodeling. The combination of these two different approaches has been previoulsy used for the study of airway remodeling in severe equine asthma [33].

Another limitation is related to the artery selection criteria, based on the external diameter, which is susceptible of being biased by the ongoing disease. Alternative methods which classify the artery size based on the length of the internal elastic lamina, have been described [30, 31]. However, in the present study, the internal elastic lamina was not always reliably outlined, despite the use of specific staining for elastic fibers, such as the Movat Russel pentachrome. As discussed above, it could be postulated that the loss of definition and integrity of the internal elastic lamina represents itself part of the remodeling process. Thus, in this study, it was not possible to apply these classification methods in a systematic way. It was postulated that any possible disease-related remodeling was more likely to be associated with an increase in the artery wall thickness (and external diameter), instead of a reduction. For this reason, a minimal artery diameter (>40 μm) was applied as an inclusion criterion, with no limitation of maximal diameter. Furthermore, a similar selection approach based on the external artery diameter has been already widely used in histomorphometric studies regarding remodeling of pulmonary arteries in human COPD [29–31, 35, 36, 50, 53].

Histological sample processing introduces technical artifacts, notably cut section that could potentially affect the morphometric analysis. As previously suggested, to overcome the problem of cut section, only arteries cut transversally were considered for the assessment (GLA/ED ratio < 3) [29]. Also, no differences were detected between groups in relation to the GLA/ED ratio, thus presence of bias due to cut section was considered unlikely in any of the studies.

In conclusion, the presence of pulmonary artery remodeling represented a new finding in equine asthma. It was possible to study the reversibility of PASM mass increase, achieved only with long-term antigen avoidance strategies, suggesting a role for inflammation in the persistence of the vascular changes. Mechanisms of PASM remodeling, endothelium and perivascular changes remains to be investigated as well as the possible alterations of distal arterioles, pulmonary capillaries and veins. Further studies should focus on the role of hypoxemia and inflammation in inducing PASM remodeling as well on its impact on pulmonary artery pressure and cardiovascular complications in severe equine asthma.

## Supporting information

S1 File (DOCX)

## References

[1] Bullone M, Lavoie JP. Asthma "of horses and men"—how can equine heaves help us better understand human asthma immunopathology and its functional consequences? Mol Immunol. 2015;66(1):97–105. 10.1016/j.molimm.2014.12.005 25547716

[2] Lavoie JP, Maghni K, Desnoyers M, Taha R, Martin JG, Hamid QA. Neutrophilic airway inflammation in horses with heaves is characterized by a Th2-type cytokine profile. Am J Respir Crit Care Med. 2001;164(8 Pt 1):1410–3. 11704587 10.1164/ajrccm.164.8.2012091

[3] Pirie RS, Collie DD, Dixon PM, McGorum BC. Inhaled endotoxin and organic dust particulates have synergistic proinflammatory effects in equine heaves (organic dust-induced asthma). Clin Exp Allergy. 2003;33(5):676–83. 10.1046/j.1365-2222.2003.01640.x 12752598

[4] Sage AM, Valberg S, Hayden DW, Firshman AM, Jacob K. Echocardiography in a horse with cor pulmonale from recurrent airway obstruction. J Vet Intern Med. 2006;20(3):694–6. 10.1892/0891-6640(2006)20[694:eiahwc]2.0.co;2 16734111

[5] Johansson AM, Gardner SY, Atkins CE, LaFevers DH, Breuhaus BA. Cardiovascular effects of acute pulmonary obstruction in horses with recurrent airway obstruction. Journal of veterinary internal medicine / American College of Veterinary Internal Medicine. 2007;21(2):302–7. 10.1892/0891-6640(2007)21[302:ceoapo]2.0.co;2 17427392

[6] Hanka J, van den Hoven R, Schwarz B. Paroxysmal atrial fibrillation and clinically reversible cor pulmonale in a horse with complicated recurrent airway obstruction. Tierarztl Prax Ausg G Grosstiere Nutztiere. 2015;43(2):109–14. 10.15653/TPG-140075 25799435

[7] Robinson NE, Derksen FJ, Olszewski MA, Buechner-Maxwell VA. The pathogenesis of chronic obstructive pulmonary disease of horses. Br Vet J. 1996;152(3):283–306. 10.1016/s0007-1935(96)80101-1 8762605

[8] Nyman G, Lindberg R, Weckner D, Bjork M, Kvart C, Persson SG, et al Pulmonary gas exchange correlated to clinical signs and lung pathology in horses with chronic bronchiolitis. Equine Vet J. 1991;23(4):253–60. 10.1111/j.2042-3306.1991.tb03713.x 1915223

[9] Sommer N, Strielkov I, Pak O, Weissmann N. Oxygen sensing and signal transduction in hypoxic pulmonary vasoconstriction. Eur Respir J. 2016;47(1):288–303. 10.1183/13993003.00945-2015 26493804

[10] Decloedt A, Borowicz H, Slowikowska M, Chiers K, van Loon G, Niedzwiedz A. Right ventricular function during acute exacerbation of severe equine asthma. Equine Vet J. 2017. 10.1111/evj.12675 28132404

[11] Dixon PM. Pulmonary artery pressures in normal horses and in horses affected with chronic obstructive pulmonary disease. Equine Vet J. 1978;10(3):195–8. 10.1111/j.2042-3306.1978.tb02260.x 567582

[12] Veyssier-Belot C, Cacoub P. Role of endothelial and smooth muscle cells in the physiopathology and treatment management of pulmonary hypertension. Cardiovasc Res. 1999;44(2):274–82. 10.1016/s0008-6363(99)00230-8 10690304

[13] Shimoda LA, Laurie SS. Vascular remodeling in pulmonary hypertension. J Mol Med (Berl). 2013;91(3):297–309. 23334338 10.1007/s00109-013-0998-0 PMC3584237

[14] Sheikh AQ, Misra A, Rosas IO, Adams RH, Greif DM. Smooth muscle cell progenitors are primed to muscularize in pulmonary hypertension. Sci Transl Med. 2015;7(308):308ra159. 10.1126/scitranslmed.aaa9712 PMC4629985 26446956

[15] Sheikh AQ, Lighthouse JK, Greif DM. Recapitulation of developing artery muscularization in pulmonary hypertension. Cell Rep. 2014;6(5):809–17. 10.1016/j.celrep.2014.01.042 24582963 PMC4015349

[16] Tormanen KR, Uller L, Persson CG, Erjefalt JS. Allergen exposure of mouse airways evokes remodeling of both bronchi and large pulmonary vessels. Am J Respir Crit Care Med. 2005;171(1):19–25. 10.1164/rccm.200406-698OC 15447945

[17] Rydell-Tormanen K, Uller L, Erjefalt JS. Allergic airway inflammation initiates long-term vascular remodeling of the pulmonary circulation. Int Arch Allergy Immunol. 2009;149(3):251–8. 10.1159/000199721 19218818

[18] Rydell-Tormanen K, Johnson JR, Fattouh R, Jordana M, Erjefalt JS. Induction of vascular remodeling in the lung by chronic house dust mite exposure. Am J Respir Cell Mol Biol. 2008;39(1):61–7. 10.1165/rcmb.2007-0441OC 18314535

[19] Harkness LM, Kanabar V, Sharma HS, Westergren-Thorsson G, Larsson-Callerfelt AK. Pulmonary vascular changes in asthma and COPD. Pulm Pharmacol Ther. 2014;29(2):144–55. 10.1016/j.pupt.2014.09.003 25316209

[20] Singh I, Ma KC, Berlin DA. Pathophysiology of Pulmonary Hypertension in Chronic Parenchymal Lung Disease. Am J Med. 2016;129(4):366–71. 10.1016/j.amjmed.2015.11.026 26706386

[21] Bullone M, Joubert P, Gagne A, Lavoie JP, Helie P. Bronchoalveolar lavage fluid neutrophilia is associated with the severity of pulmonary lesions during equine asthma exacerbations. Equine Vet J. 2018. 10.1111/evj.12806 29341228

[22] Leclere M, Lavoie-Lamoureux A, Gelinas-Lymburner E, David F, Martin JG, Lavoie JP. Effect of antigenic exposure on airway smooth muscle remodeling in an equine model of chronic asthma. Am J Respir Cell Mol Biol. 2011;45(1):181–7. 10.1165/rcmb.2010-0300OC 20935189

[23] Relave F, David F, Leclere M, Alexander K, Bussieres G, Lavoie JP, et al Evaluation of a thoracoscopic technique using ligating loops to obtain large lung biopsies in standing healthy and heaves-affected horses. Vet Surg. 2008;37(3):232–40. 10.1111/j.1532-950X.2008.00371.x 18394069

[24] Leclere M, Lavoie-Lamoureux A, Joubert P, Relave F, Setlakwe EL, Beauchamp G, et al Corticosteroids and antigen avoidance decrease airway smooth muscle mass in an equine asthma model. Am J Respir Cell Mol Biol. 2012;47(5):589–96. 10.1165/rcmb.2011-0363OC 22721832

[25] Jean D, Vrins A, Lavoie JP. Monthly, daily, and circadian variations of measurements of pulmonary mechanics in horses with chronic obstructive pulmonary disease. Am J Vet Res. 1999;60(11):1341–6. 10566805

[26] Russell HK Jr. A modification of Movat's pentachrome stain. Arch Pathol. 1972;94(2):187–91. 4114784

[27] Herszberg B, Ramos-Barbon D, Tamaoka M, Martin JG, Lavoie JP. Heaves, an asthma-like equine disease, involves airway smooth muscle remodeling. J Allergy Clin Immunol. 2006;118(2):382–8. 10.1016/j.jaci.2006.03.044 16890762

[28] Schneider CA, Rasband WS, Eliceiri KW. NIH Image to ImageJ: 25 years of image analysis. Nat Methods. 2012;9(7):671–5. 10.1038/nmeth.2089 22930834 PMC5554542

[29] Barbera JA, Riverola A, Roca J, Ramirez J, Wagner PD, Ros D, et al Pulmonary vascular abnormalities and ventilation-perfusion relationships in mild chronic obstructive pulmonary disease. Am J Respir Crit Care Med. 1994;149(2 Pt 1):423–9. 8306040 10.1164/ajrccm.149.2.8306040

[30] Fernie JM, Lamb D. Method for maximising measurements of muscular pulmonary arteries. Journal of clinical pathology. 1985;38(12):1380–7. 10.1136/jcp.38.12.1380 4078019 PMC499496

[31] Fernie JM, Lamb D. New method for measuring intimal component of pulmonary arteries. J Clin Pathol. 1985;38(12):1374–9. 10.1136/jcp.38.12.1374 4078018 PMC499495

[32] Kay JM. Comparative morphologic features of the pulmonary vasculature in mammals. The American review of respiratory disease. 1983;128(2 Pt 2):S53–7. 6881709 10.1164/arrd.1983.128.2P2.S53

[33] Bullone M, Vargas A, Elce Y, Martin JG, Lavoie JP. Fluticasone/salmeterol reduces remodelling and neutrophilic inflammation in severe equine asthma. Sci Rep. 2017;7(1):8843 10.1038/s41598-017-09414-8 28821845 PMC5562887

[34] Ochs M, Muhlfeld C. Quantitative microscopy of the lung: a problem-based approach. Part 1: basic principles of lung stereology. Am J Physiol Lung Cell Mol Physiol. 2013;305(1):L15–22. 10.1152/ajplung.00429.2012 23624789

[35] Peinado VI, Barbera JA, Ramirez J, Gomez FP, Roca J, Jover L, et al Endothelial dysfunction in pulmonary arteries of patients with mild COPD. Am J Physiol. 1998;274(6 Pt 1):L908–13. 10.1152/ajplung.1998.274.6.L908 9609729

[36] Peinado VI, Barbera JA, Abate P, Ramirez J, Roca J, Santos S, et al Inflammatory reaction in pulmonary muscular arteries of patients with mild chronic obstructive pulmonary disease. Am J Respir Crit Care Med. 1999;159(5 Pt 1):1605–11. 10228134 10.1164/ajrccm.159.5.9807059

[37] Witzenrath M, Ahrens B, Kube SM, Hocke AC, Rosseau S, Hamelmann E, et al Allergic lung inflammation induces pulmonary vascular hyperresponsiveness. Eur Respir J. 2006;28(2):370–7. 10.1183/09031936.06.00080105 16571613

[38] Hazarika S, Van Scott MR, Lust RM, Wingard CJ. Pulmonary allergic reactions impair systemic vascular relaxation in ragweed sensitive mice. Vascul Pharmacol. 2010;53(5–6):258–63. 10.1016/j.vph.2010.09.005 20888432 PMC2981655

[39] Theodorou A, Weger N, Kunke K, Rhee K, Bice D, Muggenberg B, et al Ragweed sensitization alters pulmonary vascular responses to bronchoprovocation in beagle dogs. J Appl Physiol (1985). 1997;83(3):912–7. 9292480 10.1152/jappl.1997.83.3.912

[40] Williams KJ, Robinson NE, Defeijter-Rupp H, Millerick-May M, Stack A, Hauptman J, et al Distribution of venous remodeling in exercise-induced pulmonary hemorrhage of horses follows reported blood flow distribution in the equine lung. J Appl Physiol (1985). 2013;114(7):869–78. 23372148 10.1152/japplphysiol.01170.2012

[41] Hlastala MP, Bernard SL, Erickson HH, Fedde MR, Gaughan EM, McMurphy R, et al Pulmonary blood flow distribution in standing horses is not dominated by gravity. J Appl Physiol (1985). 1996;81(3):1051–61. 8889734 10.1152/jappl.1996.81.3.1051

[42] Williams KJ, Derksen FJ, de Feijter-Rupp H, Pannirselvam RR, Steel CM, Robinson NE. Regional pulmonary veno-occlusion: a newly identified lesion of equine exercise-induced pulmonary hemorrhage. Vet Pathol. 2008;45(3):316–26. 10.1354/vp.45-3-316 18487488

[43] Stack A, Derksen FJ, Williams KJ, Robinson NE, Jackson WF. Lung region and racing affect mechanical properties of equine pulmonary microvasculature. J Appl Physiol (1985). 2014;117(4):370–6. 24925981 10.1152/japplphysiol.00314.2014

[44] Stack A, Derksen FJ, Williams KJ, Robinson NE, Jackson WF. Regional heterogeneity in the reactivity of equine small pulmonary blood vessels. J Appl Physiol (1985). 2016;120(6):599–607. 26769957 10.1152/japplphysiol.00975.2015

[45] Cook RC, Crowley CM, Hayden R, Gao M, Fedoruk L, Lichtenstein SV, et al Traction injury during minimally invasive harvesting of the saphenous vein is associated with impaired endothelial function. J Thorac Cardiovasc Surg. 2004;127(1):65–71. 10.1016/s0022-5223(03)01024-9 14752414

[46] Santos S, Peinado VI, Ramirez J, Melgosa T, Roca J, Rodriguez-Roisin R, et al Characterization of pulmonary vascular remodelling in smokers and patients with mild COPD. Eur Respir J. 2002;19(4):632–8. 10.1183/09031936.02.00245902 11998991

[47] Barbera JA, Peinado VI, Santos S. Pulmonary hypertension in chronic obstructive pulmonary disease. Eur Respir J. 2003;21(5):892–905. 10.1183/09031936.03.00115402 12765440

[48] Wright JL, Petty T, Thurlbeck WM. Analysis of the structure of the muscular pulmonary arteries in patients with pulmonary hypertension and COPD: National Institutes of Health nocturnal oxygen therapy trial. Lung. 1992;170(2):109–24. 10.1007/BF00175982 1501507

[49] Voelkel NF, Cool CD. Pulmonary vascular involvement in chronic obstructive pulmonary disease. Eur Respir J Suppl. 2003;46:28s–32s. 10.1183/09031936.03.00000503 14621104

[50] Saetta M, Di Stefano A, Rosina C, Thiene G, Fabbri LM. Quantitative structural analysis of peripheral airways and arteries in sudden fatal asthma. Am Rev Respir Dis. 1991;143(1):138–43. 10.1164/ajrccm/143.1.138 1986670

[51] Chandrasekar I, Eis A, Konduri GG. Betamethasone attenuates oxidant stress in endothelial cells from fetal lambs with persistent pulmonary hypertension. Pediatr Res. 2008;63(1):67–72. 10.1203/PDR.0b013e31815b43ee 18043518

[52] Sadowska AM, Klebe B, Germonpre P, De Backer WA. Glucocorticosteroids as antioxidants in treatment of asthma and COPD. New application for an old medication? Steroids. 2007;72(1):1–6. 10.1016/j.steroids.2006.10.007 17145070

[53] Peinado VI, Gomez FP, Barbera JA, Roman A, Angels Montero M, Ramirez J, et al Pulmonary vascular abnormalities in chronic obstructive pulmonary disease undergoing lung transplant. J Heart Lung Transplant. 2013;32(12):1262–9. 10.1016/j.healun.2013.09.007 24263025

