# Supplementary material for: Severe asthma is associated with a remodeling of the pulmonary arteries in horses
Source: PLoS One. 2020 Oct 22;15(10):e0239561. doi: 10.1371/journal.pone.0239561 (PMC7580920; doi:10.1371/journal.pone.0239561)
Supplement: S1 File — (DOCX) [file pone.0239561.s001.docx]

**Horses – study 1**

Animal age, pulmonary function results and neutrophil % on the BALF of asthmatic and controls horses. “Asthma R”, asthmatic horses in clinical remission; “Asthma E”, asthmatic horses in clinical exacerbation. Pulmonary function results are reported as lung resistance (RL), lung elastance (EL) and transpulmonary pressure (Ppl). For some horses, lung function and/or BALF were not available (NA).

| **Group** | **Age (years)** | **Lung function** | | | **BALF**  **Neutrophil %** |
| --- | --- | --- | --- | --- | --- |
|  |  | **RL**  **(cm H_2_O/L/s)** | **EL**  **(cm H_2_O/L)** | **Ppl**  **(cm H_2_O)** |  |
| Control 1 | 30 | NA | NA | NA | NA |
| Control 2 | 25 | 0.47 | 0.83 | 6.3 | 0 |
| Control 3 | 25 | 0.38 | 0.67 | 0.4 | 1 |
| Control 4 | 23 | 0.81 | 0.45 | 8.5 | 0.8 |
| Control 5 | 15 | NA | NA | NA | NA |
| Control 6 | 24 | 0.43 | 0.67 | 5.8 | 3 |
|  |  |  |  |  |  |
| Asthma R1 | 28 | 1.40 | 0.97 | 17.0 | 8.8 |
| Asthma R2 | 29 | 0.68 | 0.56 | 7.1 | 22.3 |
| Asthma R3 | 24 | 0.74 | 0.47 | 10.9 | 3.5 |
| Asthma R4 | 16 | 0.96 | 1.05 | 12.1 | 2.3 |
| Asthma R5 | 24 | 0.58 | 0.60 | 10.6 | 2.8 |
| Asthma R6 | 30 | 0.66 | 0.33 | 10.1 | 25 |
|  |  |  |  |  |  |
| Asthma E1 | 24 | 1.95 | 1.75 | 30.9 | 47.6 |
| Asthma E2 | 25 | 2.87 | 1.58 | 30.1 | NA |
| Asthma E3 | 28 | 2.91 | 1.41 | 30.7 | 37 |
| Asthma E4 | 16 | 1.75 | NA | 59.5 | 10 |
| Asthma E5 | 26 | 1.83 | 1.07 | 18.9 | 37.8 |
| Asthma E6 | 31 | 2.78 | 4.32 | 55.3 | 22.8 |

**Horses – study 2**

Animal age, pulmonary function results and neutrophil % on the BALF of asthmatic (A) and controls (C) horses. Pulmonary function results are reported as lung resistance (RL), lung elastance (EL) and transpulmonary pressure (Ppl). For asthmatic horses, data are reported during remission (R) and during exacerbation (E). The samples used for the study were collected in the remission phase. For controls, the data reported were obtained just prior to sample collection. Further data can be found as supplementary material of a previous published study about airway remodeling (1).

| **Group** | **Age (years)** | **Sex** | **Breed^a^** | **Lung function** | | | | | | **BALF Neutrophil %** | |
| --- | --- | --- | --- | --- | --- | --- | --- | --- | --- | --- | --- |
|  |  |  |  | **RL**  **(cm H_2_O/L/s)** | | **EL**  **(cm H_2_O/L)** | | **Ppl**  **(cm H_2_O)** | |  |  |
|  |  |  |  | **R** | **E** | **R** | **E** | **R** | **E** | **R** | **E** |
| A1 | 19 | Mare | STB | 0.65 | 2.39 | 0.41 | 2.39 | 6.3 | 29.5 | 0.3 | 20.3 |
| A2 | 16 | Mare | QH | 0.31 | 3.20 | 0.74 | 10.32 | 6.2 | 54.8 | 0.5 | 14 |
| A3 | 16 | Mare | STB | 0.71 | 2.60 | 0.55 | 3.69 | 6.9 | 42.8 | 0 | 6.8 |
| A4 | 15 | Gelding | AR C | 0.31 | 2.18 | 0.65 | 2.05 | 5.9 | 30.9 | 0 | 4.3 |
| A5 | 15 | Mare | CR | 0.84 | 3.24 | 0.79 | 11.93 | 8.6 | 70.9 | 7 | 54.4 |
| A6 | 20 | Gelding | AR C | 0.51 | 2.15 | 0.56 | 11.51 | 7.1 | 58.3 | 5.5 | 41.8 |
|  |  |  |  |  |  |  |  |  |  |  |  |
| C1 | 17 | Mare | STB | 0.70 | | 0.62 | | 6.8 | | 0.8 | |
| C2 | 12 | Mare | STB | 0.58 | | 0.56 | | 7.3 | | 1.3 | |
| C3 | 14 | Mare | STB | 0.24 | | 0.37 | | 4.1 | | 1 | |
| C4 | 15 | Mare | CR | 0.53 | | 0.70 | | 4.9 | | 0 | |
| C5 | 11 | Mare | STB | 0.29 | | 0.44 | | 3.8 | | 2.8 | |

**^a^Breed legend:** STB = Standardbred; QH = Quarter Horse; AR C = Arabian cross; CR = Cross.

**Horses – study 3**

Animal age, pulmonary function results and neutrophil % on the BALF of asthmatic horses treated with antigen avoidance (AA) and corticosteroids (CS). Pulmonary function results are reported as lung resistance (RL), lung elastance (EL) and transpulmonary pressure (Ppl). Data are reported during exacerbation before the study (T0) and after the 12-month treatment (T12). Further data can be found as supplementary material of a previous published study about reversibility of airway remodeling (2).

| **Group** | **Age (years)** | **Sex** | **Breed^a^** | **Lung function** | | | | | | **BALF Neutrophil %** | |
| --- | --- | --- | --- | --- | --- | --- | --- | --- | --- | --- | --- |
|  |  |  |  | **LR**  **(cm H_2_O/L/s)** | | **LE**  **(cm H_2_O/L)** | | **Ppl**  **(cm H_2_O)** | |  |  |
|  |  |  |  | **T0** | **T12** | **T0** | **T12** | **T0** | **T12** | **T0** | **T12** |
| AA1 | 16 | Gelding | AR C | 3.07 | 0.52 | 3.78 | 0.55 | 39.5 | 7.8 | 24.5 | 0.8 |
| AA2 | 17 | Mare | STB | 2.68 | 1.27 | 2.07 | 0.76 | 31.4 | 16.5 | 26.8 | 0 |
| AA3 | 13 | Mare | QH | 3.82 | 0.76 | 2.95 | 0.67 | 39.1 | 10.5 | 38.3 | 0 |
| AA4 | 23 | Mare | QH | 3.13 | 0.78 | 6.97 | 0.91 | 55.9 | 10.9 | 75 | 1.5 |
| AA5 | 18 | Gelding | QH | 3.07 | 0.98 | 4.67 | 2.03 | 52.8 | 15.4 | 19.5 | 0.5 |
|  |  |  |  |  |  |  |  |  |  |  |  |
| CS1 | 20 | Mare | CR | 4.38 | 1.04 | 4.80 | 0.87 | 51.1 | 11.8 | 41.8 | 0 |
| CS2 | 21 | Mare | QH C | 2.12 | 0.41 | 2.45 | 0.59 | 28.8 | 7.2 | 45.3 | 0.3 |
| CS3 | 16 | Mare | CR | 2.39 | 0.81 | 5.95 | 0.77 | 54.4 | 13.1 | 36.3 | 0.3 |
| CS4 | 20 | Gelding | QH | 2.97 | 0.67 | 4.60 | 0.72 | 42.8 | 9.4 | 46 | 0.3 |
| CS5 | 16 | Mare | THB C | 2.49 | 1.33 | 2.13 | 0.70 | 32.2 | 14.3 | 25.1 | 0 |
| CS6 | 19 | Gelding | QH | 2.10 | 0.54 | 3.47 | 0.33 | 48.8 | 8.0 | 63 | 1 |

**^a^Breed legend:** STB = Standardbred; QH = Quarter Horse; AR C = Arabian cross; QH C = Quarter Horse cross; THB C = Thoroughbred cross; CR = Cross

**References**

1. Leclere M, Lavoie-Lamoureux A, Gelinas-Lymburner E, David F, Martin JG, Lavoie JP. Effect of antigenic exposure on airway smooth muscle remodeling in an equine model of chronic asthma. Am J Respir Cell Mol Biol. 2011;45(1):181-7.

2. Leclere M, Lavoie-Lamoureux A, Joubert P, Relave F, Setlakwe EL, Beauchamp G, et al. Corticosteroids and antigen avoidance decrease airway smooth muscle mass in an equine asthma model. Am J Respir Cell Mol Biol. 2012;47(5):589-96.
